# Modeling and Simulation as a Tool to Assess Voriconazole Exposure in the Central Nervous System

**DOI:** 10.3390/pharmaceutics15071781

**Published:** 2023-06-21

**Authors:** Keli Jaqueline Staudt, Bruna Bernar Dias, Izabel Almeida Alves, Bénédicte Lelièvre, Jean-Philippe Bouchara, Bibiana Verlindo de Araújo

**Affiliations:** 1 Pharmaceutical Sciences Graduate Program, Federal University of Rio Grande do Sul—UFRGS, Porto Alegre 90610-000, Brazil; kelijaquelines@gmail.com (K.J.S.); b.bernardias@gmail.com (B.B.D.); 2 Faculty of Pharmacy, Federal University of Bahia—UFBA, Salvador 40170-115, Brazil; izabel.alves@ufba.br; 3 University of Angers-University of Brest, IRF (Infections Respiratoires Fongiques), SFR ICAT 4208, CEDEX 9, 49933 Angers, France; belelievre@chu-angers.fr (B.L.); jean-philippe.bouchara@univ-angers.fr (J.-P.B.)

**Keywords:** cryptococcosis, cerebrospinal fluid, voriconazole, meningitis, brain microdialysis, interstitial space fluid

## Abstract

Voriconazole is a triazole antifungal used empirically for the treatment of complicated meningitis associated with *Cryptococcus neoformans*. Biopsy studies show that the drug exhibits adequate brain penetration although levels of cerebral spinal fluid (CSF) are highly variable. Considering that CSF is one of the main surrogates for CNS exposure, the present work proposed the building of a population pharmacokinetic modeling (popPK) model able to describing the exposure achieved by voriconazole in the plasma, interstitial cerebral fluid and CSF of healthy and infected rats. The developed popPK model was described by four compartments, including total plasma, free brain and total CSF concentrations. The following PK parameters were determined: K_m_ = 4.76 mg/L, V_max_ = 1.06 mg/h, Q_1_ = 2.69 L, Q_in_ = 0.81 h^−1^ and Q_out_ = 0.63 h^−1^. Infection was a covariate in the Michaelis–Menten constant (K_m_) and intercompartmental clearance from the brain tissue compartment to central compartment (Q_out_). Simulations performed with the popPK model to determine the probability of reaching the therapeutic target of *f*AUC > MIC showed that VRC has sufficient tissue exposure in the interstitial fluid and in the CSF for the treatment of fungal infections in these tissues at prevalent minimum inhibitory concentrations.

## 1. Introduction

Cryptococcal meningitis is one of the most common fungal infections that affect the central nervous system (CNS), accounting for a large number of deaths annually [1,2,3]. The treatment of this infection comprises the use of amphotericin B (AmB), flucytosine (5-FH) and fluconazole (FLU) [4,5]. AmB has been used in the initial treatment of cryptococcosis because it is effective against *Cryptococcus* [6]; however, it has caused several side effects, mainly nephrotoxicity, which has posed a risk when used [7]. Regarding 5-FH, even though it is considered to be one of the best options due to its good penetration, fungi have developed elevated resistance with its continuous use along the years, besides its high costs and limited availability in Brazil [8,9]. An alternative to the problem of the toxicity of AmB and the resistance and high cost of 5-FH is the use of FLU; however, its use alone presents a low therapeutic success and high mortality [5,9,10].

Recently, case reports have shown that voriconazole (VRC) could be used as an alternative treatment for patients with cryptococcal meningitis whose standard treatment has failed [11,12,13]. VRC is a broad-spectrum triazole antifungal used for the treatment of patients with opportunistic fungal infections such as cryptococcosis [14]. VRC presents non-linear pharmacokinetics and high interindividual variability due to its extensive metabolism, being also an inhibitor of cytochrome P450 isoenzymes [15,16,17].

VRC presents good penetration into human brain tissue, reaching concentrations similar to or even higher than those observed in plasma. However, VRC concentrations that are observed in human cerebrospinal fluid (CSF) tend to be reduced, with CSF/plasma concentration ratios of 0.22 to 1.0 in adults [18,19,20] and 0.57 for children [21]. In rats, Alves and collaborators, using microdialysis, observed that the infection associated to *C. neoformans* caused an increase in the free brain penetration of VRC in the infected animals, with the brain exposure in terms of the ratio of the area under the concentration curve (AUC) in the brain related to plasma being 1.86 ± 1.05 for the infected group and 0.85 ± 0.22 for the healthy group [22].

In the clinical scenario, concentrations of drugs in the brain are measured in the CSF, which is the most accessible site to assess the drug extension effect. However, CSF concentrations cannot be assumed to be equal to interstitial fluid (ISF) concentrations without first investigating the distribution and concentration in these compartments due to the inherent characteristics of the barriers present in the CNS, drug-related factors and the presence of infection [23,24].

With the aim of building a single model by grouping plasma data and data from two CNS regions, this work built a POP-PK model based on plasma, ISF and CSF to understand the differences that occur in the penetration of VRC into the CNS and to allow for a better evaluation of clinical scenarios and understanding whether isolated CSF concentrations, which are used in the clinic, are sufficient predictors of drug availability in the CNS.

## 2. Materials and Methods

### 2.1. Data

The popPK model was developed using data from three preclinical studies. The study design and methodologies used to quantify VRC concentrations in the collected samples have been extensively described in those publications [22,25,26]. Briefly, the study of Li (2008) involved the evaluation of VRC concentrations in plasma from male Wistar rats who received 5 or 10 mg/kg dose of VRC intravenously (i.v.) [25]. The study performed by Alves and coworkers (2017) evaluated VRC concentrations in plasma and microdialysis samples from brain tissue of healthy and *Cryptococcus neoformans*-infected male Wistar rats, who received a 5 mg/kg dose of VRC i.v. [22]. Lelièvre and collaborators (2018) evaluated VCR concentrations in total plasma and CSF in healthy male Sprague–Dawley rats, who received a 30 mg/kg dose of VRC i.v. Total plasma and CSF samples of 3 animals for each time were collected at predefined time points after VRC administration [26].

### 2.2. Population Pharmacokinetic Analysis

Concentration–time profiles were analyzed using non-linear mixed-effects modeling in NONMEM v.7.4.3 (Icon Development Solutions, Ellifott City, MD, USA), applying the first-order condition estimation method with interaction (FOCE-I) and ADVAN13 subroutine. PsN software version 4.9.0 (Perl-speaks-NONMEM, Mats Karlsson and Rikard Nordgren, Uppsala, Sweden) and PIRANA^®^ v.3.0.0 (Pirana Software and Consulting, Certara, Princeton, NJ, USA) were used to keep track of run records and results. For graphical analysis, R program, version 4.1.1, RStudio, version 1.4.1717 (The R Foundation for Statistical Computing, Vienna, Austria) and the packages xpose4 and ggplot2 were used.

The data were modeled as log-transformed and free plasma concentrations were calculated using plasma protein binding value of 66% [22]. Microdialysate data were described by the integral over each collection interval [27]. For data analysis of VCR total plasma and CSF concentrations obtained by Lelièvre et al. (2018), we used the naive pooled data approach, which treats all observations as if they came from a single animal [28].

Structural model building was performed sequentially. First, the observed VRC plasma concentrations obtained from healthy and infected rats were fitted to a two-compartment model. The model was then expanded to include the free brain and CSF concentrations. Thus, inter-individual variability (IIV) was modeled exponentially, and residual variability was described separately for plasma, microdialysate and CSF data with a log-additive error model.

Once the base structural model had been determined, the contributions of covariates to the population parameters variability were assessed by applying a stepwise forward inclusion (*p* ˂ 0.05) and backward elimination (*p* ˂ 0.01) procedure. The infection and body weight were analyzed as covariates. Model selection was guided by significant changes in the value of the objective function (OFV), measured by at least a decrease of 3.84 or 6.64 points in OFV (*p* < 0.05 or *p* < 0.01), visual exploration of goodness-of-fit (GOF) plots and the relative standard error (RSE), informing the precision of model parameters.

In addition, for all pharmacokinetic parameters of the model, the body weight of the rats was incorporated allometrically (Equation (1)) to describe the difference between the weights of rats of different lineages from the data used [29,30].

(1) $$P_{i}=P_{TV}*\;{\left(\frac{BW_{i}}{BW_{median}}\right)}^{k}$$

In this equation, *P_i_* corresponds the individual parameter, $P_{TV}$ is the population parameter, $BW_{i}$ and $BW_{median}$ describe the individual and median body weight of the rats and k is the allometric exponent. We used k = 0.75 for maximum rate of metabolism (V_max_) and Michaelis–Menten constant (K_m_), and k = 1 for all the other parameters.

Model evaluation was performed according to GOF plots, %RSE and conditional number and through prediction-corrected visual predictive checks (pcVPCs) for each group (plasma, brain tissue and CSF). The pcVPC generated 1000 simulated profiles with its respective 10th, 50th and 90th percentiles calculated and visualized with the experimental data. A non-parametric bootstrap resampling analysis, stratified on concentration measurement site and study group with 1000 replicates, was performed to obtain the medians and confidence intervals (CIs) of the 5th and 95th quartiles, checking the model stability.

### 2.3. Simulations

Using the popPK model developed, we performed simulations to predict the human exposure to VCR in CSF and to evaluate if this exposure will be effective in treatment. To perform these simulations, we used VRC dosing regimens indicated for the treatment of fungal infections [8], allometrically scaled from humans to rats using the basal metabolic rate of each species [31]. The equations used (Equations (S1) and (S2)), resulting doses (Table S1) for simulations and the simulated concentration versus time profile (Figures S2 and S3) are presented in the Supplementary Materials. The regimens tested in the simulations were based on those reported in the literature for treating fungal infections: recommended dose—initial dose of 6 mg/kg/12 h of VRC on the first day and maintenance dose of 4 mg/kg/12 h on each subsequent day—and a dosing regimen with a 50% reduced dose—initial dose of 3 mg/kg/12 h of VRC on the first day and maintenance dose of 2 mg/kg/12 h on each subsequent day (recommended for liver cirrhosis Child–Pugh A and B) [8,32]. First, we estimated the cerebrospinal fluid (CSF)-to-plasma unbound concentration ratio (K_p,uu,CSF_) for rats by the simulation of 1000 individuals of the dataset randomly distributed. We calculated the K_p,uu,CSF_ (Equation (2)) of rats using the free plasma and free CSF exposure described by the area under the concentration–time curve (AUC) of the profiles. To calculate K_p,uu,CSF_ for humans, we corrected the value from rats with the albumin concentration in humans and rats and free plasma fraction [24].

(2) $$K_{p,uu,CSF}=\frac{f_{u,CSF\;}*\;C_{CSF}}{f_{u,plasma}*\;C_{plasma}}$$

where f_u,CSF_ represents the unbound fraction of CSF, which was calculated using Equation (3):

(3) $$f_{u,CSF}=\frac{1}{1+Q_{alb}*\left(\frac{1}{f_{u,plasma}}-1\right)}$$

where Q_alb_ is the ratio of albumin concentrations of CSF to plasma and was set to 0.005 for humans and 0.003 for rats.

The K_puu,CSF_ value calculated for rats and humans was used to estimate free concentrations in the CSF by the simulation of a model with 1000 individuals. The exposure obtained in the CSF of rats using human doses was used to predict the probability of target attainment (PTA) of treatment using a pharmacokinetic/pharmacodynamic (PK/PD) index free AUC over MIC (*f*AUC/MIC) higher than 25 [33,34] for VRC, using the EUCAST database for *C. neoformans* MIC (from 0.002 to 512 mg/L) (EUCAST, 2022). A PTA > 90% was assumed as adequate clinical outcome. So far, a PK/PD index value has not been established for voriconazole against *C. neoformans*. Therefore, we used the PK/PD ratio described for *Candida* and *Aspergillus* [33,34], assuming that the PK/PD ratio is consistent within a drug class and across pathogens [35].

## 3. Results

After a total of 287 plasma concentrations (238 from healthy rats and 49 from *Cryptococcus neoformans*-infected rats), we added 4 more healthy rats to the initial group for data enrichment, and 284 microdialysate concentrations in brain tissue (183 from healthy rats and 101 from *Cryptococcus neoformans*-infected rats) and 18 CSF concentrations were included in the population analysis. A summary table of individuals/groups and the respective number of observations is provided in Supplementary Materials (Table S2). Raw plasma and tissue concentration–time profiles for the different groups can be observed in Figure S1 of Supplementary Material.

**Figure 1:**
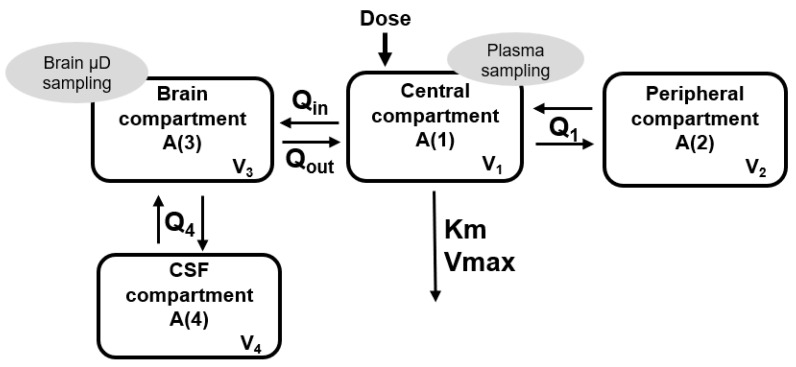
Schematic representation of the structure of the final VRC popPK model.

Plasma concentrations were best described by a two-compartment model with Michaelis–Menten (MM) elimination, parameterized in terms of V_max_, K_m_, central volume of distribution (V_1_), peripheral volume of distribution (V_2_) and intercompartmental clearance (Q_1_). The final structural model was expanded to four compartments to accommodate unbound brain concentrations and CSF concentrations. Compartments three and four describe the brain. The brain is represented as the third compartment, where microdialysate samples were collected. The volume of this compartment (V_3_) was fixed as 0.00041 L, the interstitial physiological value [36]. This compartment was linked to the central compartment with a bi-directional transport, parameterized as intercompartmental clearances in and out (Q_in_ and Q_out_, respectively). The fourth compartment, linked to the brain interstitial fluid, is represented by CSF concentrations, where we have the CSF volume of distribution (V_4_) and intercompartmental clearance (Q_4_) (Figure 1).

Equations used to estimate free concentrations at the different compartments are described below (Equations (4)–(7)):

(4) $$\frac{dAcentral\;}{dt}=-\frac{Q_{1}}{V_{1}}*A_{1\;}-\frac{V_{max}*\;A_{1}}{K_{m}+A_{1}}+\frac{Q_{1}}{V_{2}}*A_{2}+\frac{Q_{out}}{V_{3}}*A_{3}-\frac{Q_{in}}{V_{1}}\;*\;A_{1}$$

(5) $$\frac{dAperipheral}{dt}=-\frac{Q_{1}}{V_{2}}\;*\;A_{2}+\frac{Q_{1}}{V_{1}}*\;A_{1}$$

(6) $$\frac{dAbrain}{dt}=-\frac{Q_{out}}{V_{3}}*A_{3}+\frac{Q_{in}}{V_{1}}*A_{1}+\frac{Q_{4}}{V_{4}}*A_{4}-\frac{Q_{4}}{V_{3}}*A_{3}$$

(7) $$\frac{dACSF}{dt}=-\frac{Q_{4}}{V_{4}}*A_{4}+\frac{Q_{4}}{V_{3}}*A_{3}$$

where A_1_, A_2_, A_3_ and A_4_ are plasma, peripheral plasma, brain and CSF unbound amounts of VRC, respectively; V_max_ is the maximum rate of metabolism; K_m_ is the Michaelis–Menten constant; V_1_ is the central volume of distribution; V_2_ is the peripheral volume of distribution; Q_1_ is the intercompartmental clearance from the central compartment to peripheral compartment; Q_in_ is the intercompartmental clearance from the central compartment to brain tissue compartment; Q_out_ is the intercompartmental clearance from the brain tissue compartment to central compartment; V_3_ is the volume of the brain tissue compartment; Q_4_ is the intercompartmental clearance from the central compartment to CSF compartment; and V_4_ is the volume of the CSF tissue compartment. To compare observed plasma concentrations with the free concentrations estimated by the model, the estimates were divided by 0.35, the unbound fraction of the drug [22].

IIV was described by an exponential model and was then estimated for K_m_, V_1_, V_2_ and Q_out_. An additive error model for each plasma, brain and CSF concentration was sufficient to describe the residual unexplained variability. The infection was included as a covariate in K_m_ and Q_out._ The parameters K_m_ and Q_out_ were calculated separately for the infected group. The calculated K_m_ was higher in infected animals (K_m,infected_ = 8.135 (mg/L)) compared to the healthy animals (K_m_ = 4.76 (mg/L)) and the calculated Q_out_ was lower in infected animals (Q_out,infected_ = 0.388 (mg/L)) compared to the healthy animals (Q_out_ = 0.634 (mg/L)).

The final individual parameters, including the covariates, are expressed as follows:

(8) $$K_{m}=\theta_{K_{m}}*\;\left(\theta_{K_{m,infected}}\;*\;Infected\;status\right)$$

(9) $$Q_{out}=\theta_{Q_{out,infected}}\;*\;\left(\theta_{Q_{out,infected}\;}*\;Infected\;status\right)$$

where $\theta K_{m}$ and $\theta Q_{out}$ represent the typical value of the population for K_m_ and for Q_out_, respectively; $\theta K_{m,infected}$ and $\theta Q_{out,infected}$ represent the influence of infection in K_m_ and for Q_out_, respectively; and the infected status is defined as a categorical covariate with 0 and 1 for healthy and infected animals, respectively. Table 1 shows the parameters estimated by the model, with a relative standard error (%RSE) no greater than 35%, and the 95th confidence intervals from the bootstrap analysis.

**Figure 2:**
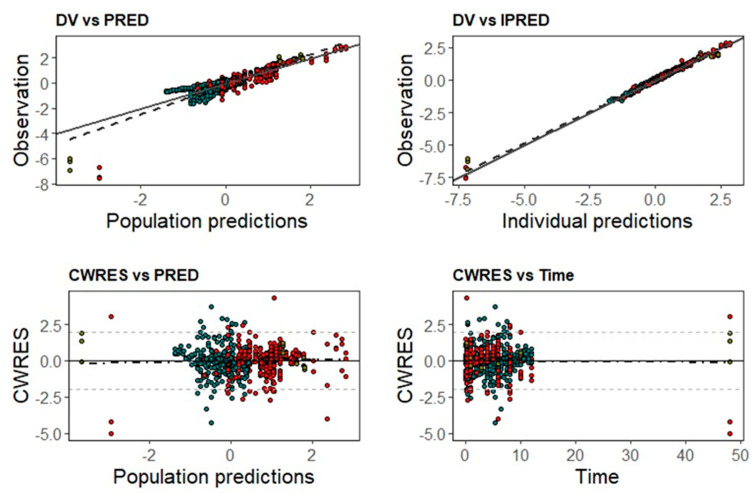
Goodness-of-fit plots from VRC final popPK showing the observations (DV) vs. population (PRED) and individual (IPRED) predictions, conditional residuals (CWRES) vs. PRED and time, in red plasma data and in green brain data.

**Figure 3:**
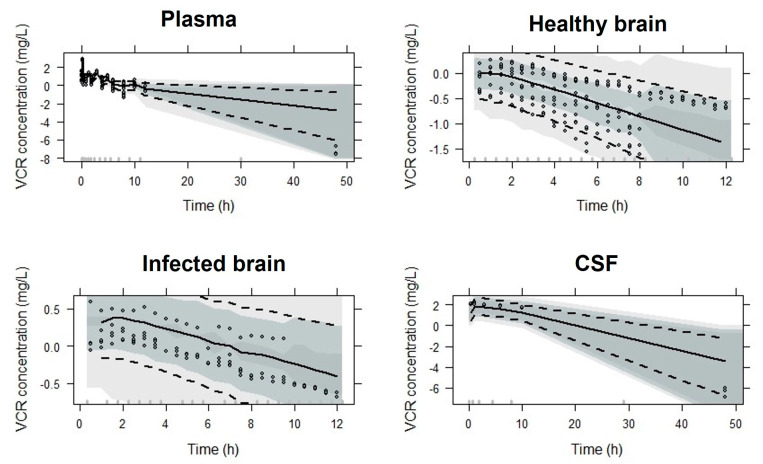
Predicted–corrected visual predictive check of the final popPK model stratified by plasma, healthy and infected brain, and CSF. VPCs are based on 1000 simulations and show a comparison of the observations (dots) with the 10th, 50th and 90th percentiles of the 1000 simulated profiles (dashed lines and shadow areas).

Model parameters were estimated with good precision and diagnostic plots showed a good agreement between the observed and predicted data (Figure 2). The pc-VPC indicated an adequate goodness-of-fit and good predictive performance of the final popPK model for all tissues investigated (Figure 3).

**Figure 4:**
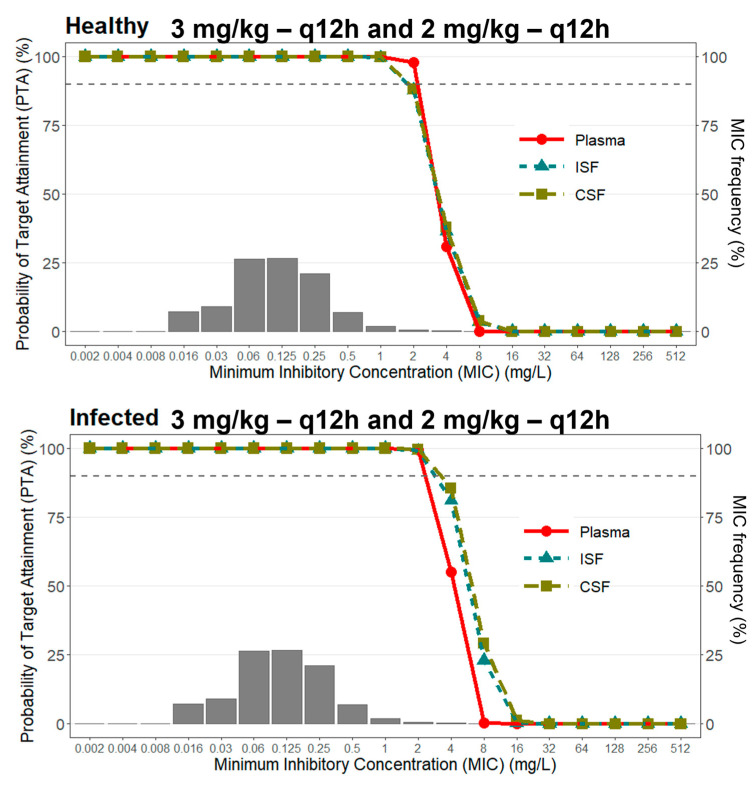

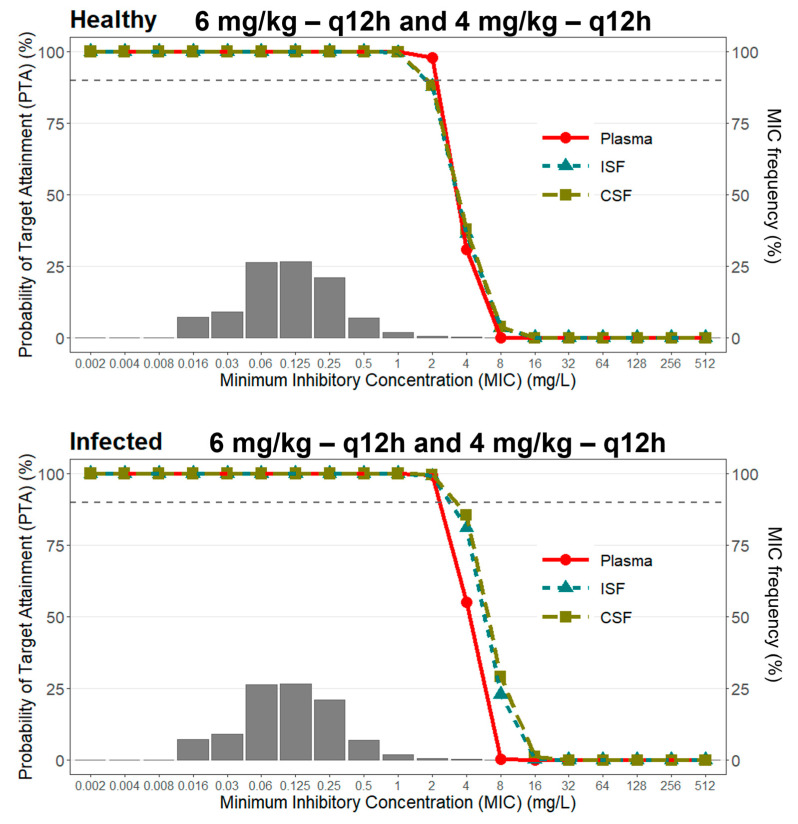
Probability of target attainment for a PK/PD index of *f*AUC/MIC > 25 for *C. neoformans* for VRC dosing regimens considering free plasma, free brain and free CSF concentrations.

To assess the PTA of dosing regimens used in clinical practice, simulations of 1000 profiles using the final popPK model were conducted to evaluate different allometrically scaled VRC dose regimens (Table S1 in Supplementary Materials). Figure 4 presents the PTA of these regimens against the MIC distribution for *C. neoformans* for free plasma, free brain and free CSF concentrations. The target PK/PD index used was a VRC *f*AUC/MIC greater than 25 [33,34] with a probability > 90%.

The PTA for the healthy and infected groups was more than 90% for the most prevalent *C. neoformans* MICs, both in plasma and tissues, when using an initial dose of 6 mg/kg/12 h of VRC on the first day and a maintenance dose of 4 mg/kg/12 h on each subsequent day [8,32]. However, from MICs > 0.5 mg/L for the healthy group and MICs > 1 mg/L for the infected group, only the free concentrations observed in the CSF show better success.

When we look at treatment with an initial dose of 3 mg/kg/12 h of VRC on the first day and a maintenance dose of 2 mg/kg/12 h on each subsequent day, which represent a 50% reduction compared to standard treatment [8,32], we observed behavior similar to that of the standard dose. However, there is a decrease from the PTA for free CSF concentrations, from an MIC of 2 mg/L in the healthy group and an MIC of 4 mg/L in the infected group, and when we look at the exposures in the brain and plasma, they are less successful.

## 4. Discussion

VRC is used for the treatment of systemic fungal infections, it being indicated for the treatment of cryptococcal meningitis in cases of resistance to other drugs, such as 5-FH, FLU and AmB. When used in monotherapy, VRC presents good activity, which can be attributed to its penetration into the CNS, including the CSF [37].

In this work, we investigated the penetration of VRC in the CNS, specifically in the ISF and CSF, developing a preclinical popPK model with data obtained from total plasma concentrations, free brain concentrations and total CSF sampling in rats. The developed model was simulated to predict free brain and free CSF exposure after VRC doses allometrically scaled from humans to rats. The PTA of free concentration–time profiles generated by model simulation for different dosing regimens to treat *C. neoformans* cryptococcal meningitis was investigated using the PK/PD index ƒAUC/MIC > 25.

The final popPK model was an extension of that previously reported for *C. neoformans* infection in brain [22], with an enrichment of data, describing information in more plasma and CSF concentrations. Differently from the previous models, the popPK model consists of four compartments (Figure 1), where the central compartment was divided into two (central and peripheral), the brain compartment was linked to the central compartment by bidirectional transport (Q_in_ and Q_out_) and the compartment CSF was linked to the brain compartment. This developed model showed alterations in the VRC distribution during the infection caused by *C. neoformans* through modifications in the pharmacokinetic parameters, such as the Michaelis–Menten constant (K_m_) and intercompartmental clearance from the brain tissue compartment to central compartment (Q_out_). The literature describes that the VRC presents non-linear pharmacokinetics due to the saturation of its metabolism from doses more than 5 mg/kg [19,22,25,26].

The calculated K_m_ was higher in infected animals (K_m,infected_ = 8.135 (mg/L)) compared to the healthy animals (K_m,healthy_ = 4.76 (mg/L)) and the value of Q_out_ was lower in infected animals (Q_out,infected_ = 0.388 (mg/L)) compared to the observed value in healthy animals (Q_out,healthy_ = 0.634 (mg/L)). One of the factors most associated with infection that can affect drug exposure in the CNS is inflammation of the meninges. This inflammation makes the BBB more permeable, in addition to causing a decrease in the return clearance for venous blood. This factor helps to explain a reduction in the Q_out_ of infected animals in relation to the Q_out_ of healthy animals. It should be noted that, during infection, concentrations are increased in the CNS; however, during treatment, they may change as patients respond, and thus inflammation may decrease, thereby lowering target site concentrations necessary for disease eradication [11].

Post hoc analysis of the area under the concentration–time curve (AUC_0–t_) was performed with predicted data obtained from the model to compare with the AUC_0–t_ of the works from which the data were taken. The results can be seen in Table S3 of Supplementary Material. All AUC values generated in the present study by the model are based on the free drug concentrations, whereas the AUC values reported in the original studies were calculated using the total drug concentrations. Thus, we multiplied these total concentration values by a free fraction of 0.34 of the VRC to obtain the free-concentration-based AUCs. A good agreement can be observed between the modeled and observed concentrations, resulting in similar AUC values to the previous studies, demonstrating that the changes in the model did not change the final conclusions of those studies [22,25,26]. Our model, in addition to plasma and brain microdialysate concentrations, encompasses more plasma concentrations and cerebrospinal fluid concentrations, which are not part of the simpler model previously reported. Thus, it has, as its main advantage, an understanding of how this VRC distribution occurs between the plasma and ISF and CSF, which are two distinct regions of the CNS that present differences between the barriers present in these places, as well as differences between the free concentrations that arrive in different regions of the CNS.

The model that we developed can help us to optimize the therapy, as it allows us to test different doses and verify whether the concentrations achieved can achieve therapeutic success. In addition, this work showed us that the CSF in the case of the VRC can be used as a predictor of the brain concentrations achieved since the concentrations achieved both in the ISF and in the CSF are similar and achieved therapeutic success in the evaluated conditions. Since the CSF is a sample widely used in clinical practice, this allows for greater ease and confidence in the use of this type of sample and in the results obtained from this analysis.

The AUC_tot_ values of VRC were higher in plasma compared to values observed in ISF. These differences reflect changes in drug brain penetration between different tissues, best demonstrated by comparing the penetration factor in plasma and the brain (*f*T_PLASMA/BRAIN_), calculated by the ratio between free AUC in plasma and in the brain, which was 0.82 for healthy mice. For infected mice, the *f*T_PLASMA/BRAIN_ was 1.50 in ISF, i.e., the infected group presented an increased VRC in brain. These results are similar to those found by Alves et al. (2017) who observed a *f*T_PLASMA/BRAIN_ of 0.85 ± 0.22 for the healthy group and 1.86 ± 1.05 for the infected group [22]. CNS infections cause an increase in BBB/CSF permeability and/or a decrease in CSF flow depending on the type of infecting microorganism or the severity of the infection, which can lead to increased drug concentrations in CNS compartments during inflammation [38]. However, CSF exposure to VRC was higher than in plasma, resulting in a *f*T_PLASMA/CSF_ of 2.57, indicating greater penetration into the CSF, agreeing with the findings by Lelièvre and collaborators, where they obtained a *f*T_PLASMA/CSF_ of 2.10 [26]. In addition, it is described in the literature that the CSF has low levels of plasma-derived proteins, with protein concentrations around 0.5% or less than those observed in plasma concentrations, which may have an impact on a greater free fraction available in the CSF, resulting in higher free concentrations [39].

CSF concentrations are used clinically as the same as or as substitutes for ISF concentrations due to the greater ease of collecting these samples in relation to ISF [11,24,39]. As far as is known, there is no restrictive barrier between the brain ISF and the CSF, so a drug that has entered the CNS can distribute fluidly between these spaces. However, it is described that this balance between concentrations does not occur in most cases due to several factors, such as drug elimination, protein binding and CSF flux [11,38].

Using the developed model, we calculated the PTA in different treatments used in clinical practice to assess the probability of these treatments reaching a pharmacological target. It is necessary to take into account that, so far, there is no established PK/PD target for VRC and *C. neoformans* and that the PK/PD target used for the analysis was the one described for *Candida* and *Aspergillus*, considering that these fungi share some similarities with *C. neoformans* [33,34]. PTA analysis of VRC dose regimens using PK/PD *f*AUC/MIC > 25 as a target showed that, for the most prevalent MICs (0.06 and 0.125 mg/L) of *C. neoformans* plasma, ISF and CSF concentrations are efficient for both healthy and infected groups. From the MIC of 4.0 mg/L for both doses, none of the observed concentrations reach PTA > 90%.

The probability of reaching the therapeutic target of *f*AUC > MIC shows that voriconazole has sufficient tissue exposure in the cerebrospinal fluid for the treatment of fungal infections in these tissues in the most prevalent MICs. The use of plasma concentrations can lead to the use of higher doses than necessary because, as we demonstrate, the CSF concentrations are higher than plasma. Furthermore, the concentrations of voriconazole observed in the CSF are shown to be predictors of the concentrations observed in the ISF; however, this information should be analyzed with caution since there is no established PK/PD index for VRC against *C. neoformans*, even though the literature describes that we can assume that the PK/PD ratio is consistent within a drug class and between pathogens.

## 5. Conclusions

In conclusion, we present the results of a popPK model of VRC concentrations in plasma, ISF and CSF. The penetration of VRC was greater in the tissue of rats infected by *C. neoformans* in relation to the healthy group, and the concentrations of free CSF showed a greater penetration in healthy animals. The PTA analysis demonstrated that the CSF concentrations obtained after an allometric dose on a scale from humans to rats are sufficient to eradicate the infection and that the CSF concentrations can be used as a predictor of the concentrations observed in the ISF in the case of voriconazole since both concentrations showed the same result in terms of reaching the PTA (>90%) in both conditions and doses tested.

## Figures and Tables

**Table 1:** Population pharmacokinetic parameters estimate for the popPK model.

Parameter	Estimate (%RSE)	Bootstrap Median (95% CI)
V_max_ (mg/h)	1.06 (13)	1.01 (0.385–1.160)
K_m_ (mg/L)	4.76 (20)	4.56 (1.128–5.993)
K_m,infected_ (mg/L)	8.13 (44)	0.68 (0.239–1.324)
Q_1_ (L)	2.69 (18)	2.64 (0.360–4.992)
V_1_ (L)	1.15 (7)	1.16 (0.953–1.469)
V_2_ (L)	0.37 (18)	0.35 (0.042–0.586)
Q_in_ (L/h)	0.81 (13)	0.83 (0.285–1.983)
Q_out_ (L/h)	0.63 (13)	0.63 (0.221–1.822)
Q_out,infected_ (L/h)	0.38 (36)	0.35 (0.117–0.549)
V_3_ (L)	0.00041FIX	-
Q_4_ (L/h)	0.33 (19)	0.35 (0.085–1.170)
V_4_ (L)	0.13 (17)	0.13 (0.049–0.310)
ωK_m_ (%CV)	38 (15)	33 (25–223)
ωV_1_ (%CV)	29 (13)	26 (15–38)
ωV_2_ (%CV)	47 (34)	52 (26–100)
ωQ_out_ (%CV)	39 (17)	37 (25–47)
Plasma log-additive error (mg/L)	0.107 (5)	0.107 (0.073–0.137)
Microdialysis log-additive error (mg/L)	0.074 (5)	0.072 (0.056–0.091)
CSF log-additive error (mg/L)	0.450 (18)	0.449 (0.239–1.324)

V_max_: maximum rate of metabolism; K_m_: Michaelis–Menten constant; V_1_: central volume of distribution; V_2_: peripheral volume of distribution; Q_1_: intercompartmental clearance from central compartment to peripheral compartment; Q_in_: intercompartmental clearance from central compartment to brain tissue compartment; Q_out_: intercompartmental clearance from brain tissue compartment to central compartment; V_3_: volume of brain tissue compartment; Q_4_: intercompartmental clearance from central compartment to cerebrospinal fluid (CSF) compartment; V_4_: volume of CSF tissue compartment; FIX: fixed value; RSE: relative standard error; CV, coefficient of variation; CI, confidence interval. Shrinkage values for ωK_m_: 5.8%; ωV_1_: 5.5%; ωV_2_: 26.7%; ωQ_out_: 20.1%.

## Data Availability

The data presented in this study are available upon request to the corresponding author.

## Supplementary Materials

The following supporting information can be downloaded at: https://www.mdpi.com/article/10.3390/pharmaceutics15071781/s1, Figure S1: Voriconazole concentration–time curves in total plasma, free brain and CSF after 5, 10 and 30 mg/kg i.v. bolus doses applied to healthy and infected *C. neoformans* groups. Points are observations; Table S1: Human dosing regimens allometrically scaled to rat, used in the simulations; Table S2: Number of animals and observations of each experimental group and condition; Figure S2: Simulated concentration versus time profile for recommended dose—initial dose of 6 mg/kg/12 h of VRC on the first day and maintenance dose of 4 mg/kg/12 h on each subsequent day, with median (line) and 32nd and 68th percentiles (shadow area) for free plasma, free brain and free CSF; Figure S3: Simulated concentration versus time profile for recommended dose—initial dose of 3 mg/kg/12 h of VRC on the first day and maintenance dose of 2 mg/kg/12 h on each subsequent day, with median (line) and 32nd and 68th percentiles (shadow area) for free plasma, free brain and free CSF; Figure S4: Individual pharmacokinetic profiles from observed data (points), populational and individual predictions (line and dashed line) for the final popPK model for voriconazole total plasma (ID 101–306: 5 mg/kg; ID 401–406: 10 mg/kg; ID 501: 30 mg/kg); Figure S5: Individual pharmacokinetic profiles from observed data (points), populational and individual predictions (line and dashed line) for the final popPK model for voriconazole-free brain after dose of 5 mg/kg i.v.; Figure S6: Individual pharmacokinetic profiles from observed data (points), populational and individual predictions (line and dashed line) for the final popPK model for voriconazole total plasma (A) and total CSF (B) after dose of 30 mg/kg i.v.; Table S3: Post hoc analysis comparing AUC_0–t_ (mg·h/L) from final popPK model and original studies.
