# Supplementary material for: Modeling and Simulation as a Tool to Assess Voriconazole Exposure in the Central Nervous System
Source: Pharmaceutics. 2023 Jun 21;15(7):1781. doi: 10.3390/pharmaceutics15071781 (PMC10384042; doi:10.3390/pharmaceutics15071781)
Supplement: Supplementary file 1 [file pharmaceutics-15-01781-s001.zip › pharmaceutics-2370621-supplementary.pdf]

Modeling and Simulation as a Tool to Assess Voriconazole Exposure in the Central Nervous System

Keli Jaqueline Staudt, Bruna Bernar Dias, Izabel Almeida Alves, Bénédicte Lelièvre, Jean-Philippe Bouchara and Bibiana Verlindo de Araújo

Supplementary Materials

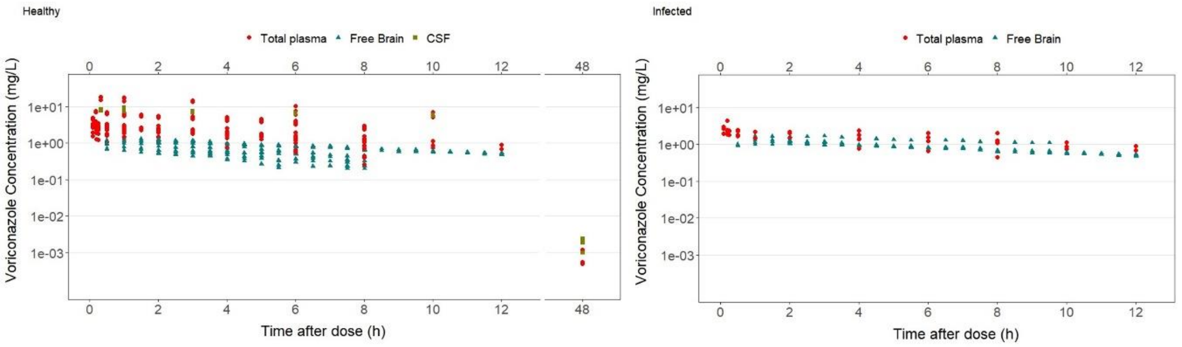

Figure S1. Voriconazole concentration-time curves in total plasma, free brain and CSF after 5, 10 and 30 mg/kg i.v. bolus doses to healthy and infected *C. neoformans* groups. Points are observations.

Equations used to perform the allometric scaling:

$$BMI = K * M^{0.75} \qquad \text{(S1)}$$

$$Dose_{rats} = \frac{Dose_{humans}}{BMI_{humans}} * BMI_{rats} \qquad \text{(S2)}$$

where BMI is the body mass index, calculated for humans and rats, with K as the proportionality constant for mean corporal temperature, which for placental mammals assumes a value of 70; M is the body mass, which assumes values of 70 kg for humans and 0.35 kg for rats.

Table S1. Human dosing regimens allometrically scaled to rat, used in the simulations**.**

Human Dose (mg/kg/12h)	Equivalent Dose in Rats (mg/kg/12h)
6.0 (first day)	22.0
4.0 (subsequent days)	15.0
3.0 (first day)	11.0
2.0 (subsequent days)	7.5

Table S2. Number of animals and observations of each experimental group and condition.

Groups	Animals/observations/status	Dose
**Plasma**		
(ALVES *et al.*, 2017)	11/100/healthy<br>5/49/infected	5 mg/kg
(LI, 2008)	6/60/healthy<br>6/60/healthy	5 mg/kg<br>10 mg/kg
(LELIÈVRE *et al.*, 2018)	21/18/ healthy	30 mg/kg
**Brain microdialysis**		
(ALVES *et al.*, 2017)	11/183/healthy<br>5/101/infected	5 mg/kg
**CSF**		
(LELIÈVRE *et al.*, 2018)	21/18/ healthy	30 mg/kg

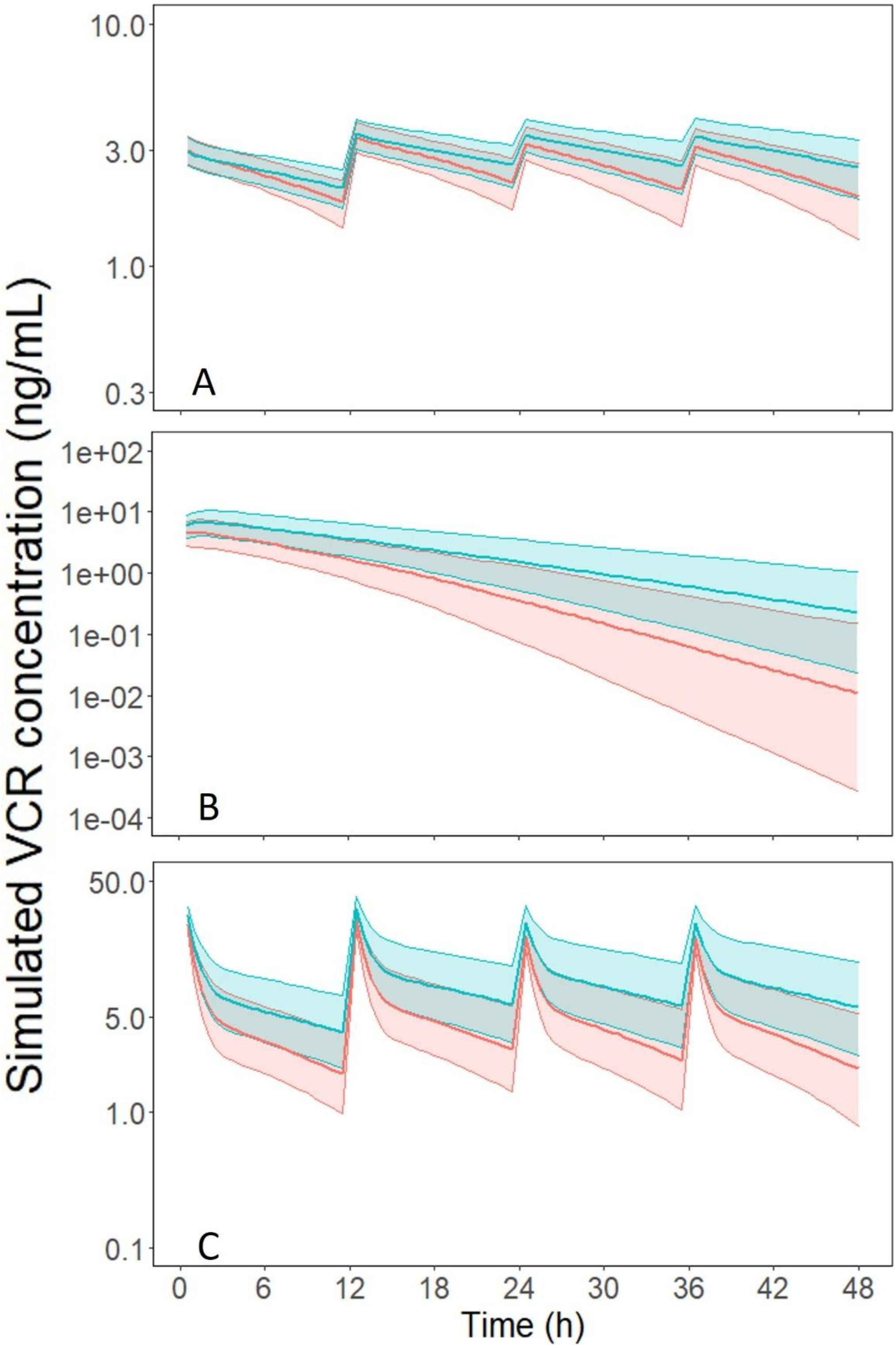

Figure S2. Simulated concentration versus time profile for recommended dose - initial dose of 6 mg/kg/12 h of VRC on the first day and maintenance dose of 4 mg/kg/12 h on each subsequent day, with median (line) and 32nd and 68th percentiles (shadow area) for A - free plasma, B - free brain, and C - free CSF (shadow area in red - healthy animals; shadow area in blue - infected animals).

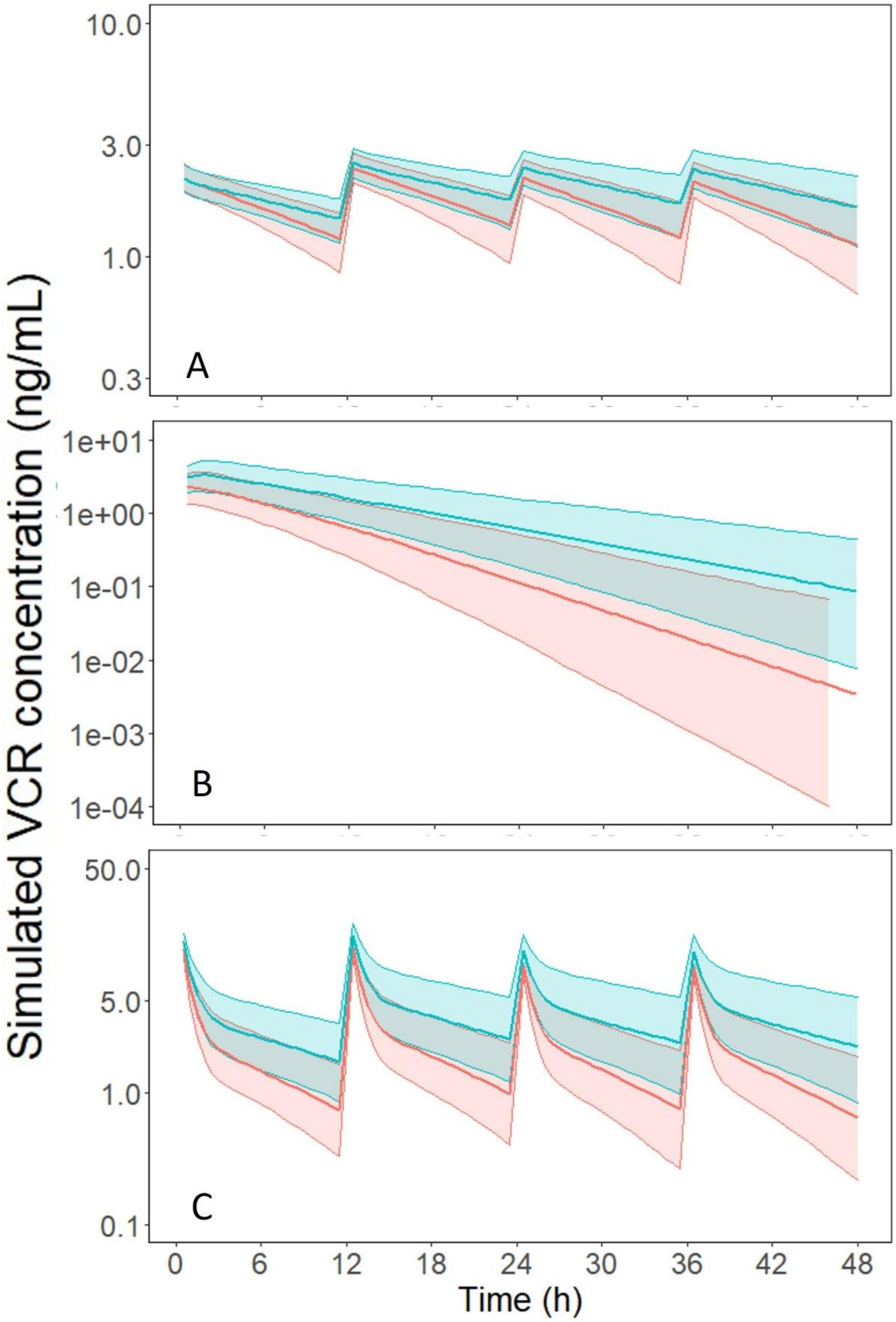

Figure S3. Simulated concentration versus time profile for recommended dose - initial dose of 3 mg/kg/12 h of VRC on the first day and maintenance dose of 2 mg/kg/12 h on each subsequent day, with median (line) and 32nd and 68th percentiles (shadow area) for A - free plasma, B - free brain, and C - free CSF (shadow area in red - healthy animals; shadow area in blue - infected animals).

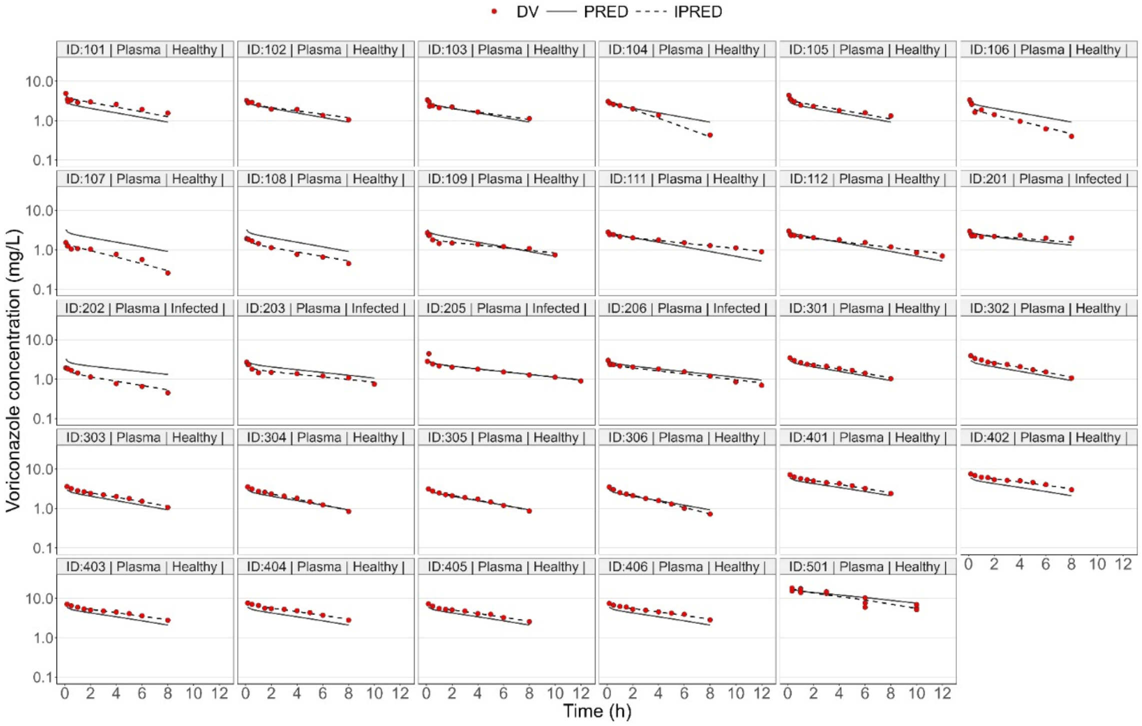

Figure S4. Individual pharmacokinetic profiles from observed data (points), populational and individual predictions (line and dashed line) for the final popPK model for voriconazole total plasma (ID 101 – 306: 5 mg/kg; ID 401 – 406: 10 mg/kg; ID 501: 30 mg/kg).

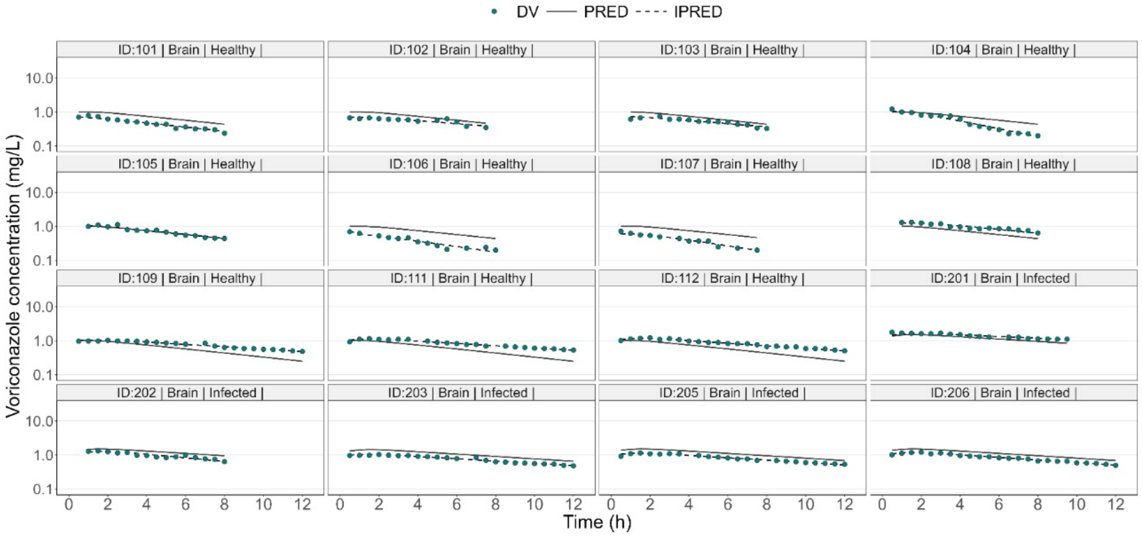

Figure S5. Individual pharmacokinetic profiles from observed data (points), populational and individual predictions (line and dashed line) for the final popPK model for voriconazole free brain after dose of 5 mg/kg i.v.

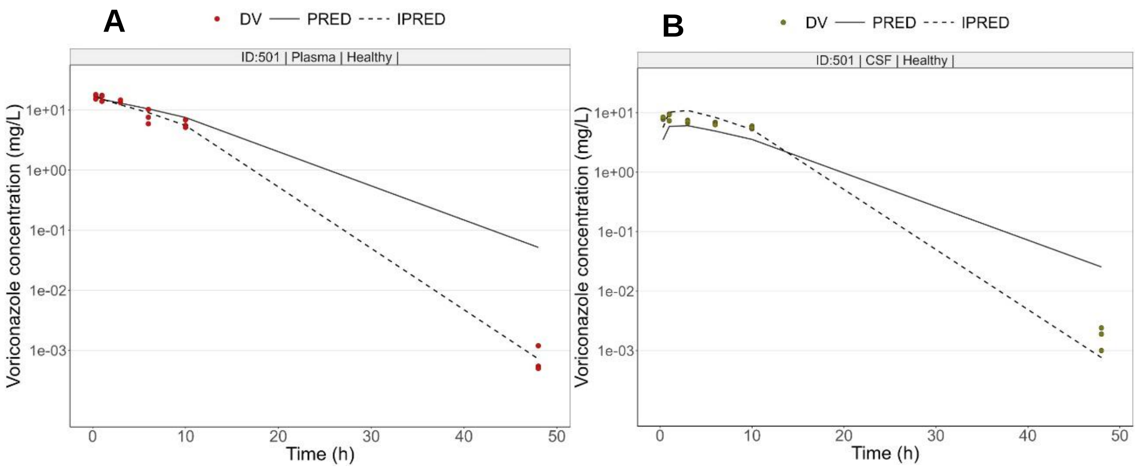

Figure S6. Individual pharmacokinetic profiles from observed data (points), populational and individual predictions (line and dashed line) for the final popPK model for voriconazole total plasma (A) and total CSF (B) after dose of 30 mg/kg i.v.

Table S3. Post-hoc analysis comparing $AUC_{0\text{-}t}$ (mg·h/L) from final popPK model and original studies.

Dose/Status/Study		$AUC_{0\text{-}t}$ free plasma	$AUC_{0\text{-}t}$ free brain	$AUC_{0\text{-}t}$ free CSF
5 mg/kg healthy	popPK model	7.31 ± 0.88	6.05 ± 2.76	na
Alves et al. 2017	Original studies	6.98 ± 2.87	5.69 ± 1.51	na
5 mg/kg infected	popPK model	7.42 ± 0.97	10. 99 ± 2.65	na
Alves et al. 2017	Original studies	8.40 ± 2.56	13.8 ± 1.53	na
5 mg/kg healthy	popPK model	8.71 ± 0.25	na	na
Li, 2008	Original studies	6.98 ± 0.87	na	na
10 mg/kg healthy	popPK model	18.78 ± 0.19	na	na
Li, 2008	Original studies	19.06 ± 1.30	na	na
30 mg/kg healthy	popPK model	121.26	na	21.36
Leliévre et al, 2018	Original studies	49.3 ± 4.48	na	103.72 ± 6.46

na: not applicable.
